# Comparison of Maxillary Molar Distalization with an Implant-Supported Distal Jet and a Traditional Tooth-Supported Distal Jet Appliance

**DOI:** 10.1155/2014/937059

**Published:** 2014-06-11

**Authors:** Mauro Cozzani, Marco Pasini, Francesco Zallio, Robert Ritucci, Sabrina Mutinelli, Laura Mazzotta, Maria Rita Giuca, Vincenzo Piras

**Affiliations:** ^1^Scientific Committee UOC Odontoiatria “G. Gaslini-Galliera” Hospital, Dipartimento di Scienze Chirurgiche, Università di Cagliari, Via Binaghi 4, 09121 Cagliari, Italy; ^2^Università di Pisa, Lungarno Pacinotti 43, 56126 Pisa, Italy; ^3^Department of Orthodontics, Università di Ferrarara, Via Montebello 31, 44121 Ferrara, Italy; ^4^Department of Orthodontics, Boston University, 100 East Newton Street, Boston, MA 02118, USA; ^5^Università di Cagliari, Via Binaghi 4, 09121 Cagliari, Italy; ^6^Department of Surgical Pathology, Medicine, Molecular and Critical Area, Università di Pisa, Lungarno Pacinotti 43, 56126 Pisa, Italy; ^7^Dipartimento di Scienze Chirurgiche, Università di Cagliari, Via Binaghi 4, 09121 Cagliari, Italy

## Abstract

*Aim*. To investigate and compare the efficiency of two appliances for molar distalization: the bone-anchored distal screw (DS) and the traditional tooth-supported distal jet (DJ) for molar distalization and anchorage loss. *Methods.* Tests (18 subjects) were treated with a DS and controls (18 subjects) were treated with a DJ. Lateral cephalograms were obtained before and at the end of molar distalization and were analysed. Shapiro Wilk test, unpaired *t*-test, and Wilcoxon rank-sum test were applied according to values distribution. The *α* level was fixed at 0.05. *Results*. Maxillary first molars were successfully distalized into a Class I relationship in all patients. The mean molar distalization and treatment time were similar in both groups. The DS group exhibited a spontaneous distalization (2.1 ± 0.9 mm) of the first premolar with control of anchorage loss, distal tipping, extrusion, and skeletal changes. *Conclusions*. The DS is an adequate compliance-free distalizing appliance that can be used safely for the correction of Class II malocclusions. In comparison to the traditional DJ, the DS enables not only a good rate of molar distalization, but also a spontaneous distalization of the first premolars.

## 1. Background

Maxillary molar distalization is a procedure normally used for correction of dental Class II malocclusions [1]. Headgears appliances were traditionally used for maxillary molar distalization but, in modern orthodontics, mechanics requiring minimal patient cooperation are more desirable both for orthodontists and patients. In addition to headgears a great number of fixed distalizing appliances such as nickel-titanium springs, magnets, pendulum, first class, fast back, and distal jet [2, 3] have been introduced: these provide an intramaxillary anchorage that does not depend on patient compliance. Often, these appliances exploit a combination of dental and palatal anchorage, together with active components, such as intramaxillary magnetic modules, pendulum springs, or loaded coil springs [4]. Fixed distalizing appliances, however, produce a reaction force on anterior teeth that may lead to anchorage loss. Moreover, at the end of molar distalization, an additional anchorage loss may occur during premolars and incisors retraction: this is not improved by bracketing additional teeth [5] and generally increases total orthodontic treatment time [3].

In addition, maxillary molars should experience a distal bodily movement, [6] without tipping, intrusion, extrusion, and rotations. However both distal tipping and distopalatal rotations can be found when distalizing molars [7].

Hence, researchers have tried to overcome the side effects of these distalizing appliances by designing new intraoral systems that involve skeletal anchorage with temporary anchorage devices (TADS), such as the bone-anchored pendulum appliance or the dual-force distalizer [8, 9].

The introduction of skeletal anchorage in orthodontics not only has allowed the simplification of many procedures conventionally employed for the control of anchorage, but also has reduced the undesirable effects of many appliances [10]. Moreover, miniscrews present many advantages, including low cost, low invasive insertion procedures, and great versatility: many authors have demonstrated that they can be used as a successful source of anchorage during orthodontic therapy [11, 12]. In addition, miniscrews can be used in children, in adolescents, and in adults for different orthodontic procedures such as distalization, retraction of maxillary anterior teeth, intrusion, and protraction of maxillary posterior teeth and remain almost stationary throughout orthodontic loading, if they have been correctly positioned [11, 13].

In this study the distal screw (DS) has been used for treatment: it is a skeletally anchored version of the distal jet (DJ) that has two palatally applied miniscrews for a bone-supported anchorage [14].

To our knowledge, a comparison of maxillary molar distalization with an implant-supported distal jet and a traditional tooth-supported distal jet appliance has not been previously described in literature. The aim of this study, therefore, is to thoroughly investigate the clinical effects of a new bone-anchored appliance (DS) in comparison to the traditional tooth-supported appliance (DJ), for molar distalization and anchorage loss.

## 2. Materials and Methods 

Inclusion criteria for this study were patients who presented a mixed or permanent dentition and a bilateral full cuspid angle Class II molar relationship, without any transverse or vertical discrepancies. Subjects that showed poor oral hygiene and motivation together with presence of erupted second molars before distalization were excluded from the sample.

The final sample consisted of 36 consecutively treated patients; 18 patients were treated with the DS (test group: *n* = 18; 8 males and 10 females with a mean age of 11.5 ± 1.7 years), while 18 patients, that rejected the DS option, were treated with the DJ (control group: *n* = 18; 10 males and 8 females with a mean age of 11.2 ± 1.3 years).

The distal jet (American Orthodontics, Sheboygan, WI, USA) is an intramaxillary palatal appliance, which exerts its effects via a compressed nickel-titanium (Ni-Ti) coil spring between the banded maxillary first molars and the Nance button. Banded first premolars are also connected to the Nance button, premolar anchor unit.

The distal screw (Micerium, Avegno, Italy) has 2 miniscrews inserted in paramedian position in the palatal area between the first premolars and the first molars; they are enclosed in a metal plate, which is supported by a Nance button, for additional anchorage [15]. The Nance button is also used for additional support to help drive back the anterior teeth, after the molars have been actively distalized with the DJ or DS appliance.

**Figure 1:**
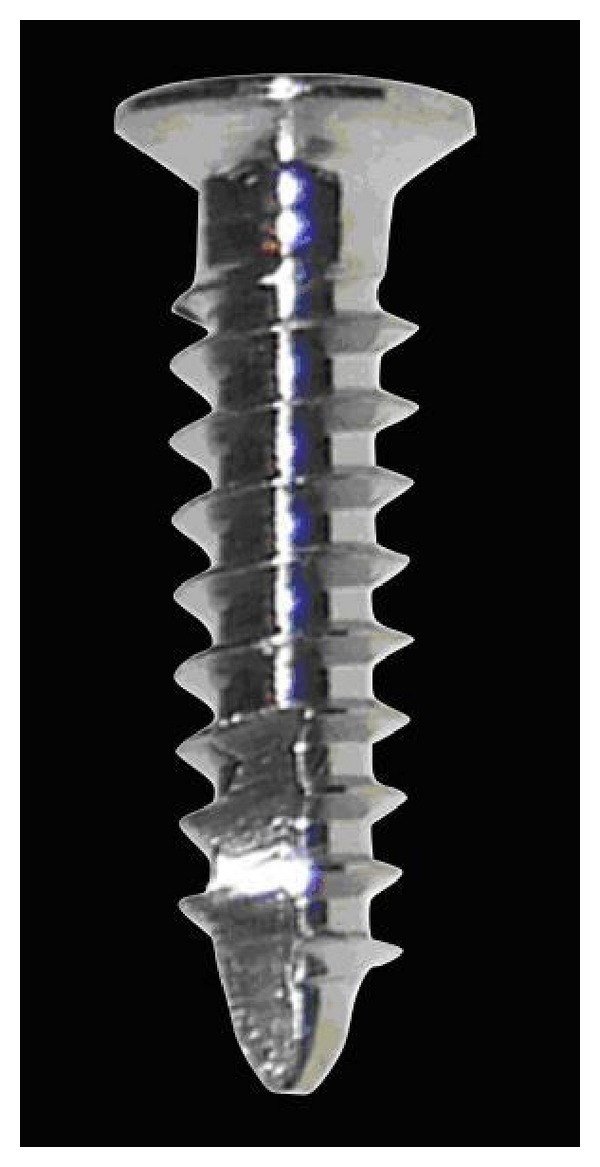
M.A.S. Miniscrew, used for the DS.

The miniscrews used (M.A.S., Micerium, Avegno, Italy) were in titanium, 11 mm long, and shaped like a truncated cone with a diameter of 1.5 mm at the point and of 2.2 mm at the neck (Figure 1). The shank of the screws was 1 mm in diameter and the threaded part had a length of 8 mm. The heads featured a hexagonal slot to house the head of the screwdriver or the contra-angle handpiece. Prior to screw placement the palatal area was locally anaesthetised and the patient rinsed with a 0.1% chlorhexidine gluconate solution. Predrilling was performed and the miniscrews were inserted by means of a manual screwdriver. Superelastic Ni-Ti coil springs with a force of 240 cN were compressed to achieve the force needed for distalization. Additional reactivations were carried out at 1 month intervals. Distalization continued until Class II molar relationship was overcorrected to a bilateral super Class I molar relationship. The appliance was then inactivated and left in place as a retention device.

Despite the fact that all subjects were strictly encouraged to maintain their oral hygiene, some plaque accumulation was present under the Nance button. This condition determined a slight palatal soft tissue irritation only in 1 DS patient; however, after the patient rinsed with a 0.1% chlorhexidine solution for 1 week, the irritation disappeared.

**Figure 2:**
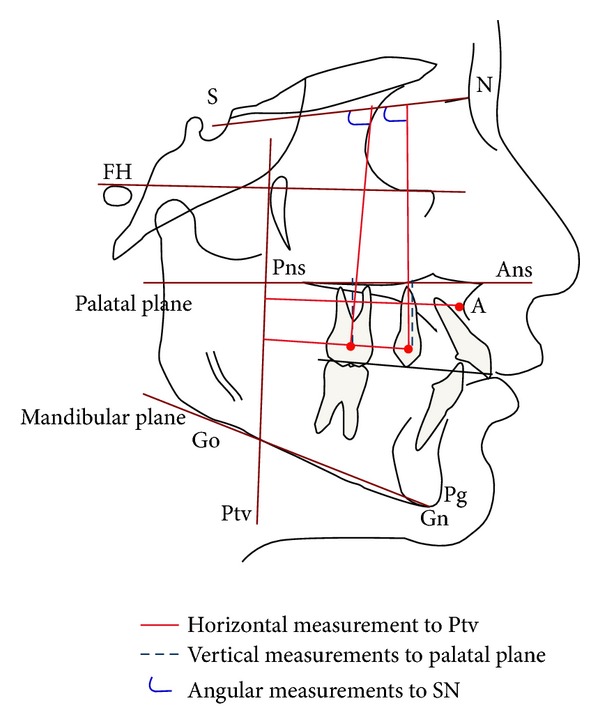
Cephalometric analysis.

Lateral cephalograms before treatment and at the end of molar distalization were acquired and measured; changes in the two groups were analyzed to determine the dental and skeletal effects, according to the methodology suggested by different authors [16, 17] and similar to the one proposed by Ghosh and Nanda [18] (Figure 2); this enabled to determine sagittal changes of the upper maxilla and vertical, sagittal, and angular changes of the first molars and first premolars. The variables considered are reported in Table 1.

All cephalometric tracings and measurements were performed by the same researcher. Furthermore, all the variables were measured twice, with a 1 week interval between the 2 registrations in order to apply Dahlberg's formula [19]: the method error resulted less than 1 mm for linear measurements and 1.5° for angular measurements.

## 3. Statistical Analysis

Some of the variables (PTV-U6, SN-U4, PP-U6, and PTV-A) were normally distributed (Shapiro Wilk test, *P* > 0.05), hence the significance in mean differences was estimated with unpaired *t*-test.

The parametric *t*-test was replaced with two-sample Wilcoxon rank-sum test, when the variables did not follow a normal distribution (SN-U6, SN-U4, and PP-U4; Shapiro Wilk test, *P* < 0.05). The *α* level was fixed at 0.05.

## 4. Results

Maxillary first molars were successfully distalized into an overcorrected Class I relationship in all patients. The mean distalization time was 9.1 ± 2.8 months in the DS group, in comparison to the DJ group 10.5 ± 4.2 months; no significant difference was found in treatment duration between the two groups. Statistical analysis did not present significant differences between male subjects and female subjects, in relation to all parameters examined in the present study. Age-adjustment was not performed, because of the very similar values between the groups. During the study 6 miniscrews became mobile and had to be replaced.

Cephalometric data and results are shown in Table 2. Maxillary first premolars (PTV-U4) distalized spontaneously 2.1 ± 1.8 mm in patients treated with DS, while, in the patients treated with DJ, it slightly mesialized 0.9 ± 1.6 mm and the difference between the two groups was statistically significant (*P* = 0.001).

**Figure 3:**
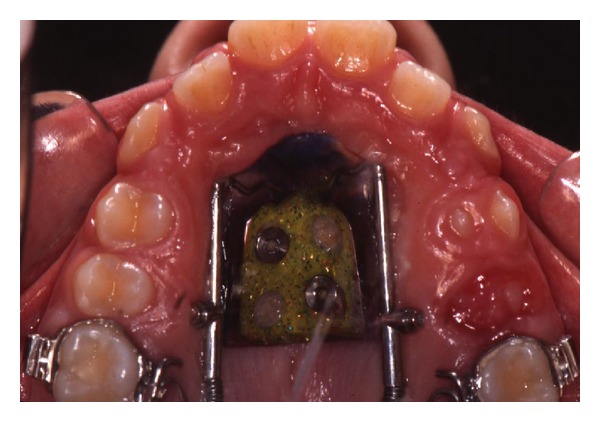
Distal screw at the beginning of treatment.

**Figure 4:**
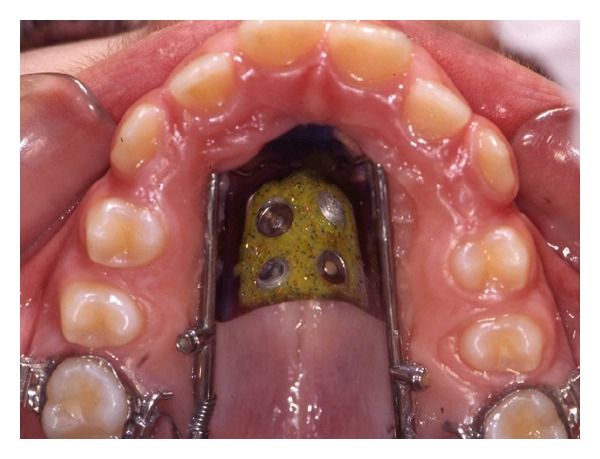
Distal screw at the end of distalization.

**Figure 5:**
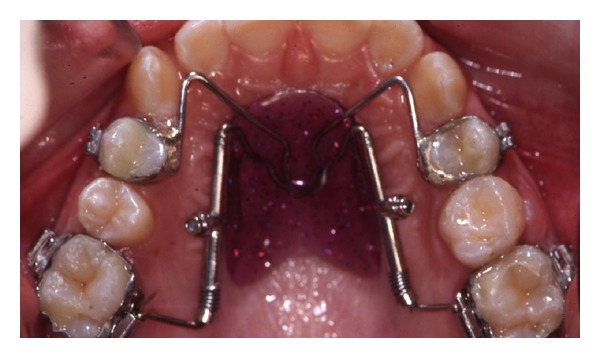
Distal jet at the beginning of treatment.

**Figure 6:**
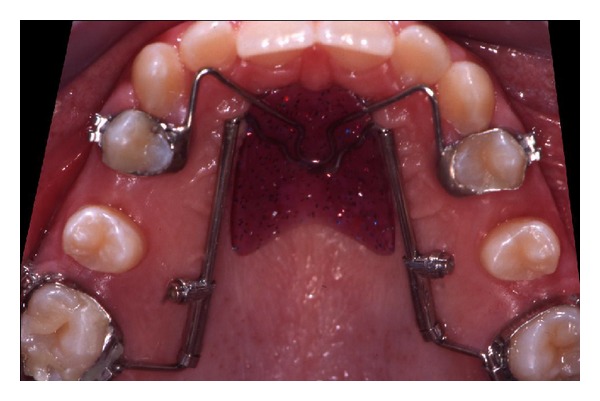
Distal jet at the end of distalization.

Maxillary first molars (PTV-U6) distalized on average 4.7 ± 1.6 mm in the test group (Figures 3 and 4) and 4.4 ± 2.5 mm in the controls (Figures 5 and 6); difference between the groups was not statistically significant. Moreover, at the end of treatment, maxillary first molar distal tipping (SN-U6) results were slightly lower in the DS group (−2.8°; −3.1 to 1.3) in comparison to the DJ group (−5.0; −9.0 to 2.0), however the difference was negligible. Variations in maxillary first premolars distal tipping (SN-U4) results also were not significant being −3.0° (−3.7 to 1.3) in the test group and −1.0° (−4.8 to 0.8) in the control group.

Molar extrusion, with respect to the maxillary plane (PP-U6), was similar between the two groups (0.7 ± 1.9 mm in the DS group and 0.4 ± 2.5 mm in the DJ group; not significant). Maxillary first premolars instead (PP-U4) presented a lower extrusion in the DS group (1.1 mm; 0.1 to 1.9) in comparison to the controls (3.5 mm; 1.0 to 4.0) and the difference was statistically significant (*P* = 0.0364).

Finally, maxillary position (PTV-A) was stable in both groups (0.4 ± 0.7 mm in the DS subjects and 1.1 ± 2.4 mm in DJ subjects; not significant).

## 5. Discussion

Findings of the present study showed a relative equivalency between DJ and DS. In fact both appliances proved to be valid clinical options for distalization of maxillary first molars: the degree of upper first molar distalization was similar and a super Class I molar relationship was achieved in the treatment time in both groups. Moreover no statistical significant differences were found in molar or premolar distal tipping, molar extrusion and maxillary position.

However, a significant spontaneous first premolar distalization was observed in the DS group, while in the DJ group first premolars slightly mesialized: this may be explained by the fact that the distal jet appliance is bonded to first premolars; on the contrary, in the distal screw premolars are not bonded and are therefore pulled by transeptal fibers in a more distal position [20]. The premolar distal movement is spontaneous, hence it might contribute to a decrease of orthodontic treatment time and to a fewer use of devices such as elastic chains or coil springs, used to distalize this tooth [21]. Also, premolar extrusion was different in the two groups since maxillary premolars in the DS group significantly extruded more compared to premolars in the DJ group: this is explained by the fact that DS patients do not have premolars bonded.

Comparing these data with that of Kircelli et al. [22], where a bone-anchored pendulum was used, we have noted that a pendulum is able to achieve a greater molar distalization (6.4 ± 1.3 mm) in a shorter period (7 ± 1.8 months) and a greater extent of spontaneous first premolar distalization (3.8 ± 1.1 mm). However, the patients treated with a pendulum appliance present a degree of molar and premolar distal tipping that is considerably higher (9.1 ± 4.6 degree and 7.7 ± 5.1 degree, resp.). These findings were similar to those of Polat-Ozsoy et al. [8], that analysed dentoalveolar and skeletal effects obtained with 2 types of pendulum appliances with different anchorage designs (bone anchored versus conventional appliance). Also it is important to take into consideration Hilgers' study [23] and the results by Byloff et al. [24, 25] and by Kinzinger et al. [26] who obtained 6.07° and 3.07°–4.75° in terms of distal tipping with a pendulum appliance.

Oberti et al. [9], investigating the clinical effects of a bone-supported distalizing appliance, called dual-force distalizer and achieved a higher molar distalization (5.9 ± 1.7 mm) in a shorter time (5 months), but the average molar inclination was higher (5.6 ± 3.7 degree).

Similar to our study, Kinzinger et al. [13] achieved upper molar distalization with a skeletal anchored distal jet, anchored to the first premolars with 2 palatal miniscrews, but without the inclusion of the Nance button. However, in contrast to our results, a mesial first premolar movement of 0.72 mm was recorded, maybe due to the lack of the application of the Nance button, which allows a better dispersion of orthodontic forces.

Also, another study investigating the efficiency of distal screw [14] showed very similar results to the ones in this study, a mean molar distalization of 4.7 mm in 9.1 months and 2.1 mm of premolar distal movement.

Moreover, the studies by Bolla et al. [27] and by Ngantung et al. [28] showed that the distal jet alone is an efficient appliance for correction of class II malocclusion; however the distalizing force on the maxillary molar results in 71% molar distalization and 29% reciprocal anchorage loss measured at the maxillary first premolar.

Chiu et al. [29] compared the effects of a distal jet and the effects of a pendulum appliance: the pendulum subjects demonstrated significantly more distal molar movement and less anchorage loss at both the premolars and the maxillary incisors than did the distal jet group. However, both appliances induced the same amount of correction in molar relationships.

For what concerns the use of miniscrews, studies from Escobar et al. [30] and Gelgor et al. [31] have shown that bone anchored appliances are more efficient in controlling anterior anchorage loss and in decreasing treatment time.

Our sample consisted of patients with a mean age of 11.5 ± 1.7 years: literature shows that the degree of difficulty and prognosis when distalizing appears to be related to the stage of dental development and to the age of the patient. The highest success rate with the fewest complications occurs when molars are moved distally in the mixed dentition stage of development [32]: it would be interesting to compare the effects of DJ and DS in adults with full eruption of second molars.

Better anchorage control and a slight spontaneous first premolar distalization make the DS a more effective molar distalization device, in comparison to traditional intraoral appliances; moreover the DS simplifies the treatment from an operative point of view since premolar banding is rendered unnecessary and the same appliance, once inactivated, can further be employed as an anchorage for final premolar and canine retraction. Moreover, it has been demonstrated in literature that the palate area is a safe zone for application of TADS [33, 34] also in children and adolescent, as long as the miniscrew is positioned in the paramedian area to prevent possible developmental disturbances of the midpalatal sutures [35–37], since the transverse growth of the midpalatal suture continues up to the late teens and is not fused completely even in adults [38, 39]. Radiographic verification of screw placement and surgical guides are therefore no longer needed and miniscrews can be inserted directly through the modified Nance button, which acts as a guide, when the appliance is cemented.

## 6. Conclusions

DS is a compliance-free distalizing appliance that can be used safely for the correction of Class II malocclusions. In comparison to the traditional DJ, the DS enables not only maxillary molar distalization, with a good rate of movement, but also a spontaneous distalization of first premolars that may decrease the total treatment time.

## Figures and Tables

**Table 1:** Cephalometric variables.

Variable	Description
PTV-U6 (mm)	Horizontal measurement from maxillary first molar to PTV line
PTV-U4 (mm)	Horizontal measurement from maxillary first premolar to PTV line
SN-U6 (degrees)	Angle formed by the axis of the maxillary first molar and the SN line
SN-U4 (degrees)	Angle formed by the axis of the maxillary first premolar and the SN line
PP-U6 (mm)	Vertical measurement from maxillary first molar to palatal plane (PP)
PP-U4 (mm)	Vertical measurement from maxillary first premolar to palatal plane (PP)
PTV-A	Horizontal measurement from point A to PTV line

**Table 2:** Statistic analysis.

Variable	DS group T1	DJ group T1	DS group T2-T1	DS group T2-T1	*P* value
PTV-U6					
Mean (SD), mm	20 (2.1)	20.2 (2.8)	−4.7 (1.6)	−4.4 (2.5)	0.6373*
PTV-U4					
Mean (SD), mm	36.2 (1.8)	37.2 (2.9)	−2.1 (1.8)	0.9 (1.6)	<0.001*
SN-U6					
Median (Iqr^†^), degrees	68 (66 to 72.8)	68 (66 to 70.8)	−2.8 (−3.1 to −1.3)	−5.0 (−9.0 to −2.0)	0.0815^‡^
SN-U4					
Median (Iqr^†^), degrees	82.5 (76.0 to 84.5)	80.5 (77.5 to 84.8)	−3.0 (−3.7 to −1.3)	−1.0 (−4.8 to 0.8)	0.5793^‡^
PP-U6					
Mean (SD), mm	14 (1.2)	15.5 (1.7)	0.7 (1.9)	0.4 (2.5)	0.6951*
PP-U4					
Median (Iqr^†^), mm	17.5 (16.3 to 19)	19 (17.3 to 20)	1.1 (0.1 to 1.9)	3.5 (1.0 to 4.0)	0.0364^‡^
PTV-A					
Mean (SD), mm	48.7 (3.3)	47.9 (2.9)	0.4 (0.7)	1.1 (2.4)	0.2316*

*Unpaired *t*-test, *α* = 0.05.

^†^Iqr is interquartile range.

^‡^Two-sample Wilcoxon rank-sum test, *α* = 0.05.
